# Quiet Revolution by SMEs in the midstream of value chains in developing regions: wholesale markets, wholesalers, logistics, and processing

**DOI:** 10.1007/s12571-021-01224-1

**Published:** 2021-10-13

**Authors:** Thomas Reardon, Lenis Saweda O. Liverpool-Tasie, Bart Minten

**Affiliations:** 1 Michigan State University, East Lansing, MI USA; 2 International Food Policy Research Institute, Yangon, Myanmar

**Keywords:** Small and medium enterprises (SMEs), Food value chains, Food processing, Logistics, Wholesale markets, Small farms, Africa, Asia, Latin America

## Abstract

Small and medium enterprises (SMEs) in the midstream (processors, wholesalers and wholesale markets, and logistics) segments of transforming value chains have proliferated rapidly over the past several decades in Africa, Asia, and Latin America. Their spread has been most rapid in the long transitional stage between the traditional and modern stages, when value chains grow long and developed with urbanization but are still fragmented, before consolidation. Most of Sub-Saharan Africa and South Asia, and parts of the other regions, are in that stage. The midstream SMEs in output and input value chains are important to overall food security (moving about 65% of food consumed in Africa and South Asia), and to employment, farmers, poor consumers, and the environment. The midstream of value chains is neglected in the national and international debates as the “missing middle.” We found that it is indeed not missing but rather hidden from the debate, hence “the hidden middle.” The midstream SMEs grow quickly and succeed where enabling conditions are present. Our main policy recommendations are to support the SMEs further growth through a focus on infrastructure investment, in particular on wholesale markets and roads, a reduction of policy-related constraints such as excessive red tape, and regulation for food safety and good commercial practices.

## Introduction

Agrifood value chains (AVCs) have been growing and transforming rapidly over the past three decades in developing regions. The transformation has been “pulled” by urbanization, liberalization, privatization, and income growth, and facilitated by investments of millions of small and medium enterprises (SMEs)^1^ as well as by investments by large foreign and domestic firms (Reardon et al., 2019). We focus on a "Quiet Revolution" by the SMEs in the “midstream” of AVCs, comprising the wholesale, logistics, and processing segments. Much of Latin America, Central and Eastern Europe, and Southeast and East Asia have started in the past several decades the “modern revolution” stage of AVC transformation. However, most of Sub-Saharan Africa (SSA) and South Asia are mainly in the transitional stage in which SMEs dominate the AVCs and large enterprises (LEs) are just emerging; we thus focus most on those two regions.

The midstream SMEs are crucial to food security, defined by FAO (2006) as including food availability, access, utilization, and stability. We show that midstream SMEs play a crucial role in: (1) food availability (levels, affordability, and stability over time) by assuring the functioning of food output AVCs and farm input AVCs that then fuel farm productivity; (2) food access in an inclusive way, indirectly by providing the inputs to own-farming, and directly by employing a fifth to a quarter of rural and urban full time equivalents (FTEs) and providing the mainstay of cash to buy food; (3) food utilization of consumers, conditioned by the investments and behavior of midstream SMEs that condition food quality and safety.

From observation of the national and international policy debates and the research literature we believe that food security policy debates neglect midstream SMEs despite their importance to food security. We focus on these SMEs to help redress that neglect. When midstream SMEs are evoked in international and national policy debates, they are often referred to as stagnant, traditional, severely constrained, part of a “missing middle.” Instead, we find evidence of rapid proliferation and dynamism of midstream SMEs and counter the term “missing middle” with our term the “hidden middle.” The latter indicates that the vibrant midstream segments of SMEs exist but have been relatively neglected in or hidden from the debate. We also contend that midstream SMEs do not need inventing, as that would be “reinventing the wheel;” rather, their further development should be encouraged and their constraints diminished so that they can grow faster and further yet.

In this paper, in Sect. 2 we sketch the stages of transformation of AVCs as general context of midstream SME development. We then analyze evidence concerning the patterns in the evolution of wholesale markets and wholesale and logistics SMEs in Sect. 3, and processing SMEs in Sect. 4. Section 5 examines channels of impacts of midstream SMEs on farmers, consumers, the environment, and employment. Section 6 concludes.

We draw on two sources of information: (1) we review other authors’ survey-based studies of the midstream SME sector; (2) we summarize findings from our own extensive primary surveys on midstream SMEs (and farm households and retailers interacting with them) in Asia and Africa in the past 15 years. We call these “stacked surveys.” The latter comprised a set or “stack” of surveys of representative samples of actors in each segment of the AVC of a product. Table 1 shows the set of our 37 primary stacked surveys, reported in 41 studies mainly from Africa and South and Southeast Asia (and some from Latin America) in the past 15 years, with an overall sample of 10,400 midstream SMEs, 5700 retailers, and 17,600 small-medium farmers. Most of the product AVCs were from main production zones to primary or secondary cities. While the surveys were not usually nationally representative, they depict the basic characteristics of the rural–urban AVCs for those products, and the midstream SMEs in them. We believe our review of these surveys represents the broadest examination in the literature to date of SMEs in the midstream of AVCs in these two regions.

**Table 1:** List of primary sources of survey data on the midstream as well as retailers and farms in value chains in SSA, Asia, and Latin America

Country	Products	Midstream		Farm	Retail	Authors (year)
			Africa			
Bangladesh	Fish, feed	950		1925	134	Hernández et al. (2018, 2021)
Bangladesh	Rice	110		220	140	Minten et al. (2013b), Reardon et al. (2012, 2014)
Bangladesh	Potato	110		220	140	Reardon et al. (2012)
Brazil	Tomato, lettuce	15		55	33	Mainville & Reardon, (2007)
China	Rice	60		250	160	Reardon et al. (2012, 2014)
China	Potato	57		320	170	Reardon et al. (2012)
Ethiopia	Teff	515		1200	282	Minten et al. (2016a)
Ethiopia	Wheat, maize, teff	71		-	-	Minten et al. (2014a)
Ethiopia	Milk	102		955	208	Minten et al. (2020b)
Ethiopia	Veg	56		810	-	Minten et al. (2020a)
Guatemala	Tomato	-		164	-	Hernández et al. (2007)
India	Fruit/veg	2200		1600	-	Fafchamps et al. (2008)
India	Potato	27		256	-	Minten et al. (2014b)
India	Potato	149		270	1079	Das Gupta et al. (2010), Reardon et al. (2012)
India	Rice, wheat, veg	78		810	190	Reardon et al. (2011a)
India	Soya, wheat	86		810	172	Reardon et al. (2011b)
India	Rice, chilies	83		810	101	Rao et al. (2011)
India	Nuts	60		217	150	Minten et al. (2013a)
India	Rice	115		270	650	Reardon et al. (2012, 2014)
India	Vegetables	480		-	-	Minten et al. (2010)
Indonesia	Mangoes	150		404	-	Qanti et al. (2017)
Indonesia	Shrimp	200		1000	-	Yi & Reardon, (2015); Yi et al. (2018)
Indonesia	Tomato	-		600	-	Hernández et al. (2015b)
Mexico	Guava	-		298	-	Hernández et al. (2015a, 2015b)
Mexico	strawberries	65		302	-	Berdegué et al. (2008)
Mexico	Fruits, veg	71		-	-	Echánove & Reardon, (2006)
Myanmar	fish	140		87		Belton et al. (2018a, 2018b)
Myanmar	Rice	122			-	Belton et al. (2018b)
Nigeria	Fish, feed	289		224	-	Gona et al. (2018)
Nigeria	Fish, feed	290		121	124	Ebiloma et al. (2018)
Nigeria	Maize, feed, chicken	1447		1478	582	Liverpool-Tasie et al. (2017, 2020b); Liverpool-Tasie & Parkhi, (2020)
Nigeria	Maize	315		-	-	Sanou et al. (2021)
Philippines	Mangoes	139		252	-	Dela Cruz et al. (2010)
Tanzania	maize	85		-	234	Alphonce et al. (2019)
Tanzania	Maize	313		-	1163	Snyder (2018)
Tanzania	Fruits, veg	15		-	-	Ijumba et al. (2021)
Uganda	Milk	800		1600	-	Van Campenhout et al. (2021)
Zambia	Milk	43		420	-	Neven et al. (2017)
**Total**		**10,395**		**17,603**	**5,712**	**33,710**

## The stages of transformation of AVCs as context for the evolution of midstream SMEs

There has been a lot of variation in the timing of take-off and speed of transformation of AVCs across products, regions, countries within regions, and zones within countries. In general, the transformation is over three stages of change in structure and conduct. We summarize here from Reardon et al. (2019) and Reardon and Minten (2020).

The “traditional” stage of AVC transformation is no longer common in Africa, Asia, and Latin America, and persists mainly in hinterland areas. AVCs in this stage tend to: (1) be spatially short (“local”); (2) be fragmented in structure (dominated by micro-enterprises, the smallest SMEs); (3) use technologies intensive in labor but with little capital; (4) have exchange arrangements characterized by “spot markets,” that is, without contracts; (5) not have formal standards.

The “transitional” stage is now strongly dominant in most of SSA and South Asia, but has reduced to half or less of the food economies of Southeast and East Asia and Latin America. The AVCs in this stage tend to: (1) be spatially long (as cities grow and their market catchment area is larger and larger, so that their food procurement reaches deeper and deeper into rural areas); (2) be fragmented in structure, highly dominated by SMEs with only the bare emergence of LEs (large enterprises); (3) feature emerging disintermediation such as by urban wholesalers supplanting use of rural brokers with third-party logistics (3PLS) SMEs; (4) use mainly labor-intensive but increasingly capital-using technologies; (5) still mainly use “spot markets;” (6) have emerging public standards of quality (such as milk or fruit grades) in the emerging formal sector.

The “modern” stage is now perhaps roughly at least half of the food economies of Southeast and East Asia and Latin America, and just emerging with perhaps about a fifth to a quarter of the food economies of Africa and South Asia. The AVCs in this stage tend to: (1) be spatially long; (2) be consolidating in various segments (such as in retail, the rise of supermarkets and large processors) and multi-nationalizing via FDI (foreign direct investment); (3) see a concomitant trend in the reduction of the share of SMEs in many segments as LEs outcompete them mainly due to economies of scale; (4) feature some “dis-intermediation” such as supermarkets buying directly from processors and processors from farmers; (5) feature capital intensification as the modern stage which tends to coincide with higher labor costs; (6) see the emergence of private standards and some use of contracts.

## Wholesale markets and wholesale/logistics SMEs

### Urbanization driving wholesale sector structural transformation over 50 years

A key driver of the diffusion and transformation of wholesale markets and wholesale SMEs has been urbanization of developing regions over the past five decades,^2^ coupled with road system development. The increase in urbanization started earliest in LAC and developing East Asia, then in South Asia, and most recently in SSA. Structural change in the wholesale sector also developed over regions roughly in that sequence, with Africa for example today in the midst of wholesale sector changes that LAC and developing East Asia experienced several decades ago. There were four broad correlations that occurred over the 50 years as a function of urbanization, as follows.

#### Correlation of urbanization and road development with the size of the wholesale sector and the spread of wholesale markets

The wholesale sector expanded rapidly over the past 50 years along with urbanization. Wholesaling expanded in and between rural areas and cities as AVCs lengthened geographically. It also expanded in volume to supply the rapidly growing food volumes demanded by cities (for example, 800% in volume of rural–urban supply chains in African in 25 years, Haggblade, 2011). In these regions, rural–urban supply chains reached deeper and deeper into rural areas to feed expanding cities (as occurred also for London in the 1500 s-1700s per Braudel, 1979). This spurred proliferation of village traders or rural brokers as well as wholesalers operating in rural towns (for teff in Ethiopia see Vandercasteelen et al., 2018).

The wholesale sector also expanded inside cities. During the transitional stage the expansion mainly took place via the spread of wholesale markets. In the late transitional and modern stages the expansion shifted to being increasingly outside the wholesale markets; we turn to this latter below.

In the traditional stage of AVC transformation, village periodic markets (such as the haats in India) and rural brokers were dominant because the great majority of the population was rural. Emerging cities were supplied grain, animal products, and fruits and vegetables by peri-urban farmers and by rural brokers gathering in small “truck markets” (unregulated open air wholesale markets or trader/trucker clusters on the outskirts of cities).

In the transitional stage, as cities started to grow rapidly, municipal and national governments worried about the ability of small, scattered truck markets to feed cities. They worried about urban food security’s reliance on informal grain traders, especially as then, governments and researchers saw traders as unreliable (given to speculation), exploitative, and having high marketing margins (Reardon & Timmer, 2007).

Governments, often supported by international donors, took two steps to address those worries (Reardon & Timmer, 2007). First, they set up grain parastatals as the legal channel for grain procurement and sales, and as a way of controlling and subsidizing urban food prices. The problems of the parastatals are well documented, such as generating large fiscal deficits, often not having a pro-poor bias in subsidy distribution, and supplying only a small share of the overall grain market leaving the rest to be supplied by informal, parallel trade. These problems, combined with the rise of Structural Adjustment Programs in the late 1980s and 1990s, led to at least partial liberalization and privatization of the parastatals in many countries and a return to grain wholesale by private traders (see Kherallah et al., 2002 for SSA and Rashid et al., 2008 for Asia).

Second, municipal and national governments established regulated wholesale markets in primary cities for both grains and perishables such as produce and fish. The objective was to regulate, license, and tax wholesalers and force rural to urban informal trade to channel into central wholesale markets (e.g., for SSA, Tollens, 1997; for India, Fafchamps et al., 2008; for China, Ahmadi-Esfahani & Locke, 1998). Selling to truck markets on the urban fringes and to unregulated wholesale markets in primary cities was in many countries declared illegal, such as in Tanzania, although only partially enforced (Lynch, 1999).

Third, as primary cities grew, the regulated central wholesale markets soon were outgrown, congested, and sometimes dilapidated due to under-investment in maintenance and expansion. Two reactions occurred in what appears to have been a common sequence across regions.

On one hand, the congested central markets were increasingly avoided by wholesalers wanting to avoid taxes and have more client options. Lynch (1999) showed in his 1994 survey of the Dar es Salaam regulated central market that from its founding in 1973 to 1993 it had gone from a near monopoly (de jure and de facto) to only 50% of the city’s intake of produce. The rest was undertaken in a handful of informal markets dispersed inside the fast-expanding city. By 2021, Dar es Salaam is a city of at least 5 million (15 times larger than it was in 1973), and has spread over a large area, engulfing many villages that had been peri-urban horticulture areas and/or former truck markets serving Dar. Now there are at least 15 produce wholesale markets (most of them formal in that they are managed by the municipality but not official in the sense of being the only legal channel) and the original “official market” is but a small part of total wholesale (Ijumba et al., 2021).

On the other hand, the congested old central wholesale market was sometimes moved outside of the center of cities to open areas near the cities and highways, such as in Mexico City in the 1990s, Beijing in the 2000s, and Addis Ababa in the 2010s. Sometimes, as in Hangzhou in the 2010s, the central wholesale market and other markets that had emerged were consolidated and moved outside the city.

Fourth, wholesale markets were also set up by municipal governments in secondary and tertiary cities (e.g., in Latin America, Reardon, 2016). These cities have been emerging rapidly in all three regions, growing up from villages and conurbations of small towns that “graduated” into small cities, such as occurred rapidly in Latin America in the 1990s (Berdegué et al., 2015) and Nigeria over the 1990s-2000s (Bloch et al., 2015).

Fifth, in the 1990s-2000s, permanent wholesale markets spread quickly in rural towns, especially in Asia. This occurred by two paths.

On one hand, in the 1970s-1990s there was a widespread proliferation of rural periodic (weekly or bi-weekly) markets^3^ and then, for some subset (the size of which is unknown due to a lack of information) there was a gradual transformation into permanent markets of some rural periodic markets in villages that grew into rural towns and tertiary cities as urbanization picked up in the 1980s-1990s. But such transitions to permanency had often started earlier for periodic markets that were located on main roads with continuous traffic and trade, nodes that themselves were the anchors for the growth of towns (Hay, 1977). Hay (1977) used the example of Fiditi north of Ibadan in Nigeria; in the 1970s it was a main road crossing with a rural market; today it is a city of 70,000 and is in the path of expansion of the primary city Ibadan which absorbs smaller towns and cities into it.^4^

On the other hand, governments, especially in Asia, invested extensively in public wholesale markets in rural towns coupled with asphalting rural roads in the 1990s and 2000s. In China, Huang et al. (2007) illustrate this for tomato and cucumber production areas in Shandong. They show that introduction of these markets had important effects on farming in the market catchment areas of the small cities and rural towns by reducing transaction costs of farmers reaching local markets. This increased adoption of vegetable farming and production intensification. In Bangladesh, the government made extensive investments in fish wholesale markets in rural areas; Hu et al. (2019) showed that these served as nodes for the formation of wholesale and logistics SME clusters across fish farming areas which encouraged and facilitated commercialization, intensification, and species diversification in fish farming.

Where there were similar road and wholesale market investments in secondary and tertiary cities and rural towns in SSA, such as in Ethiopia, there was induced proliferation of wholesale and logistics SMEs (e.g., in the teff and other cereal supply chains), large aggregate investments by these SMEs in for example truck size increases, and subsequent reduction of marketing margins and transport costs despite the removal of fuel subsidies. Similar to China and Bangladesh, the development of these spontaneous clusters of wholesale and logistics SMEs induced farmers to upgrade to better varieties of teff, use more fertilizer, and market more (Minten et al. (2014a, 2016a) and Vandercasteelen et al. (2018)).

#### Correlation of urbanization with the concentration across urban wholesale markets and among wholesale SMEs, along with the rise of 3PLS

With urbanization there has been two categories of wholesale concentration. First, there has been concentration among wholesale markets subsequent to the initial proliferation of wholesale markets inside cities, especially in Southeast/East Asia, Latin America and South Africa, as most of South Asia and SSA are still in the earlier sub-phase.

For example, Huang et al. (2007) found that there had been a proliferation of wholesale markets for produce in Beijing in the 1990s, but by 2005 many markets had exited (during a decade of extremely rapid increase in Beijing’s consumption of produce) and produce wholesale was concentrated in just a few large wholesale markets. Xinfadi, the largest by 2005, went from wholesaling one-third of Beijing’s produce in the early 1990s to wholesaling the majority by 2005, moving 8000 tons of vegetables and receiving 1000 trucks per day, to become the largest wholesale market in Asia. Similar intra-wholesale market sector consolidations occurred in other countries in Asia and Latin America, such as Guatemala and Chile, where Hernández et al. (2007) and Dirven and Faiguenbaum (2008) showed the leading wholesale markets having reached 85% and 60% shares of produce wholesale in Guatemala City and Santiago.

Second, however, the degree of consolidation among wholesale SMEs within wholesale markets varies a lot over countries and products. On one extreme one finds situations, such as in produce wholesale in Beijing in the mid-2000s, where most of the wholesalers in the markets remained quite small (employing 2–6 persons) despite consolidation over wholesale markets (Huang et al., 2007). The other extreme appears mainly to be in grains which may have economies of scale in trading, where many of the wholesale SMEs tend to be medium sized. For instance, in Nigeria, Liverpool-Tasie et al. (2017) found that an average urban maize wholesaler in Northern Nigeria (the main maize region) sold 735 tons per year, representing the output of about 370 small farmers; the average trader employed 5.4 permanent persons; the Gini coefficient over wholesalers' sales in those northern markets was 65%, hence quite concentrated; but in the non-maize zone in the south which mainly receives maize from the north, the average urban maize trader scale was 10 times smaller in yearly volumes moved, and the employee average only 1.6.

Moreover, there are cases, especially in the later transitional and early modern stages, where strong concentration occurs even in produce wholesale markets. For example, in a study of the huge Mexico City main produce wholesale market during 1987–1992, in the case of 11 main fruits and vegetables, only 91 wholesalers, forming 4% of wholesalers in those products, sold 76% of the total volumes (Echánove, 2002). Large threshold investments in inter-seasonal distribution networks were increasingly necessary for wholesalers: this requirement was gradually driving out the smaller traders.

Third, there are many case studies showing that the scale of wholesale SMEs is increasing because of “disintermediation” by urban wholesalers, especially in the mid to late transitional stage. Urban wholesalers in secondary and tertiary cities in production zones are no longer mainly sourcing via village/rural traders. Instead, they are increasingly buying directly from farmers and using third-party logistics (3PLS) to fetch the produce from the farms. Evidence of this shift has been found in situations as diverse as maize trading in Nigeria (Liverpool-Tasie et al., 2017) and rice and potato trading in India (Reardon et al., 2012).

This trend has started earlier and gone further in the more developed zones of a given country. For example in India, the disintermediation has occurred more in the dynamic states with rapidly growing cities, such as in Western and Central Uttar Pradesh, and less in lagging, hinterland areas such as Eastern Uttar Pradesh (Reardon et al., 2011a, b). There is substantial evidence of this disintermediation, for example in Indonesian horticultural AVCs (Natawidjadja et al., 2007), and rice AVCs in Bangladesh, China, India, and Vietnam (Reardon et al., 2014).

#### Correlation of urbanization and the role of 3PLS SMEs

In the traditional and early transition stages, wholesalers and processors have in-house logistics, such as their own vehicle and storage area, as distances are short and volumes are low. In the later transitional and early modern stages, when distances are large and volumes are large, the 3PLS (third party logistics) segment takes off as firms in the other segments outsource logistics rather than acquire and manage their own large trucks and warehouses. In the later modern stage, often LEs in the food industry invest in in-house capacity in logistics.

3PLS SMEs have played two sets of roles supporting wholesale. On the one hand, 3PLS replaced the need for SME wholesalers to undertake the threshold investments of transport and storage. In Nigeria, maize wholesale markets were built in consumption zones in the South and production zones in the North and consumption cities in the North, creating a network of wholesale markets stretching 1000 km from North to South. In this network, thousands of wholesale SMEs move 75% of their maize via 3PLS trucking firms and obtain most of their storage by renting 3PLS warehouses (Liverpool et al., 2017).

On the other hand, 3PLS firms have themselves substantially transformed AVCs directly and at times dramatically. For example, in South Asia, there has been a recent and rapid rise of potato cold storage SMEs in Bangladesh (Reardon et al., 2012) and in India (in Bihar, Minten et al., 2014a, b; and in western Uttar Pradesh in Agra (where the Taj Mahal is located) near Delhi, Das Gupta et al., 2010). We focus on the Agra case. The study showed that a confluence of factors led to a rapid diffusion of cold storages: (1) the development of vegetable demand in Delhi; (2) the improvement of the road link from Agra to Delhi; (3) the introduction of a disease-resistant and long-shelf-life potato variety; (4) the introduction of an electricity grid; (5) the partial subsidizing of irrigation pumps and cold storage equipment; and (6) the economy’s generating investable funds among companies in the secondary city that were partly invested in cold storages.

The diffusion of use of modern cold storages was rapid: in the early 1990s few farmers grew potatoes in Agra and there were almost no modern cold storages. By the late 1990s cold storages had risen to store 40% of the vastly larger potato output, and by 2009, 80%. Traditional on-farm storage went from ubiquitous in the early 1990s to just 1% of the potato harvest by 2009. Delhi went from sharply seasonal potato consumption (from the fresh harvest) to multi-season availability and 65% of consumption from cold-storage potatoes, mainly from Agra. Rural brokers were sidelined as the cold storages themselves became the main locus of intermediation, with urban wholesalers coming (despite formal regulations barring this) to buy potatoes from farmers at the storages.

#### Inverted-U curve relating the share of the wholesale markets in the wholesale sector with urbanization

In the early modern stage, the share of wholesale markets in the overall wholesale sector gradually drops. For example, in Mexico there is evidence of a fall in wholesale market volumes in the first half of the 2000s. The estimates include a 25–30% drop in produce sales from the Mexico City wholesale market, and 40 and 50% reductions in the Guadalajara and Monterrey markets over 2002–2005 (Reardon et al., 2007); UCMAG and CECIC (2005) reported a slow rise in the sales of the Monterrey wholesale market from 1993 to 2002 of 14%, and then a rapid fall of 55% in 2003.

This decline appears to be due to two trends. First, food retailers start to source directly from processors and farms, and processors, directly from farmers. This tends to occur where the suppliers are medium or large firms so that risk and transaction costs are low for the buyer. Examples include supermarket chains buying directly from fruit agribusinesses in Central America (Berdegué et al., 2005), and supermarkets in Beijing buying directly from large rice mills and those mills directly from medium-sized farmers (Reardon et al., 2014).

Second, food industry firms gradually leave off mainly sourcing from wholesale markets and relying increasingly on off-market “specialized dedicated wholesalers” (Reardon & Berdegué, 2002). These firms work directly with farmers and supermarkets and food service chains. Examples include Pedraza for tomatoes and chilies in Mexico (Echánove & Reardon, 2006) and Bimandiri sourcing mangoes for supermarket chains in Indonesia (Natawidjaja et al., 2007). This trend also occurs in South Africa. For example, Freshmark started as an SME specialized wholesaler of produce in 1987 and was then purchased by Shoprite (a large supermarket chain) to be its procurement arm. Freshmark expanded along with Shoprite multinationally in Africa and became a large enterprise (subsidiary of Shoprite) with its own system of distribution centers and refrigerated logistics.

These wholesalers tend to be medium sized SMEs in developing regions, and even large firms (like Sysco in the US) in developed countries where the share of the wholesale market has become minimal in retail and food service sourcing. The food industry firms thus reduce their transaction costs and outsource the enforcement of their private standards of quality and sometimes safety.

### Transformation of the conduct of wholesale/logistics SMEs

Often one observes in food security debates a sharp distinction between “traditional” and “modern” enterprises. But abundant evidence is emerging that there is a category between these poles, an intermediate or transitional type of SME that has transformed and innovated beyond being traditional, but is not fully modern, and supplies local domestic markets; this point echoes this contention for SMEs in Reardon et al. (2012) for rice and potatoes in Bangladesh, China, and India, in Minten et al. (2013a) for nuts in India, and for horticulture in Rwanda in Verhofstadt and Maertens (2013). This mirrors at a micro level the shift from traditional to transitional stages of AVC transformation discussed above. SMEs in wholesale/logistics need to shift from traditional to non-traditional, transitional forms of conduct to adapt to rapidly changing market circumstances and needs of their suppliers and their buyers – and to compete with emerging large enterprises. Below we treat these shifts.

#### Demise of “tied output-credit” arrangements between SME wholesalers and farmers

In the traditional setting, farmers had little rural nonfarm employment (RNFE) or other cash sources and so relied on wholesalers for advances. These “tied credit-output market” (Bardhan, 1980) arrangements locked in the farmers to sell to the trader at harvest time. This led to the “exploitative trader hypothesis” that in turn gave an impetus to the formation of the parastatals, at least for grains, to obviate the traders (Reardon & Timmer, 2007).

However, there is substantial recent evidence that wholesalers’ provision of advances to farmers has declined substantially. Adjognon et al. (2017) used farm household data from LSMS surveys in African countries to show that few farmers received advances from wholesalers, in kind (such as inputs) or in cash. Reardon et al. (2014) analyzed data from stacked surveys on rice in China, India, and Bangladesh (noted in Table 1), and showed with triangulated data that very few farmers received advances from wholesalers and few wholesalers provided advances. However, they found that a substantial share of wholesalers received advances from retailers and processors, but these advances were just for a one-week transaction cycle.

The reasons given in these studies for the demise of the arrangement were that: (1) rural households had other sources of cash, especially RNFE (rural nonfarm employment); (2) the improvement in roads and the proliferation of traders meant competition was keen and farmers “side sold” regardless of the tying. It is interesting that the exceptions to the demise include traders giving advances to large farmers, such as in Myanmar in aquaculture (Belton et al., 2018a, b), as traders considered this a good investment in reducing transaction costs to obtain larger volumes at one go.

#### Investment by and competition among wholesalers leading to lower margins and marketing costs in AVCs

It is traditional in the literature on traders to assert the existence of high margins and lack of competition. This has been challenged in two ways. First, in the debate about privatizing parastatals in the 1980s, various studies (such as Scott, 1985 for potato wholesalers in Peru) noted that margins of traders were high not because of exploitative behavior but because they faced, and passed on, high transaction costs and risk.

Second, in the 1990s and after, it has been contended in several studies that wholesale margins are declining due to investments by and competition among wholesalers. Competition in wholesaling appears to reduce wholesalers’ margins in the transitional compared with the traditional stage. The evidence is scarce but some studies such as Minten and Kyle (1999) note that traders’ margins in the hinterland are higher due to poor roads and high transport costs to the hinterland zones, than in the more competitive and lower transaction cost peri-urban and intermediate rural zones. This suggests that margins are lower in this stage due to cost and competition.

Moreover, Minten et al. (2014a) showed in Ethiopia that as SME traders and truckers invested over the past decade and proliferated: (1) margins, i.e., price gaps between farms and consumers were reduced, as the market became more efficient; (2) spatial integration over the countries wholesale markets increased; (3) mill and retail margins reduced; and (4) traders proliferated and competition increased sharply.

#### Product and price differentiation by wholesalers

One often observes the claim in the farm marketing literature that wholesalers traditionally do not “reward” farmers for variety and quality differentiation, that they buy unsorted product from the farmers and then sort and sell differentiated products to the market, capturing quality differentiation rents. We have found mixed evidence on this.

On the one hand, it appears to still be the case in situations where it is a “buyers’ market,” where farmers face few market options, where farmers adopted the crop variety for production cost and not market differentiation reasons, or where rewards for quality are too small to differentiate. The first is illustrated by Hernández et al. (2015) for guavas in Mexico; wholesalers paid quality premia to farmers in the zone near roads and markets where wholesalers competed for guava supply but did not pay quality-differentiated prices in hinterland areas. The second is illustrated by Minten et al. (2013b) for a new higher quality variety of rice in Bangladesh; wholesalers did not pay farmers more for that variety than for others but wholesalers received higher prices from mills for that new variety. The third is shown by Anissa et al. (2021) who find in Ethiopia that wheat markets reflect a ‘market of lemons’ where wheat supply is bulked and mixed but that the cost of third-party certification is too high to incentivize quality differentiation at the farm level.

On the other hand, wholesalers pay quality-differentiated prices, even in local domestic markets, where they are competing for supply (such as in the advanced guava zone in Mexico) or where they must reward farmers for practices that are costly in labor or capital, that the traders would have to do if they could not induce the farmers to do for them. An example is maize wholesalers in Nigeria paying farmers differential prices for dried (ready for milling) versus humid maize (Liverpool-Tasie et al., 2017).

#### Bundling other services with wholesaling

Wholesale SMEs often "bundle" services beyond the narrow service of intermediation per se (arranging a purchase or sale and paying or being paid). The services fit the needs of the seller or buyer in the face of "idiosyncratic market constraints" that impede their clients from getting those services otherwise.

Wholesale SMEs help small farmers indirectly by establishing resource-provision relations with farms that bring farmers services such as logistics and inputs and credit. These complementary services are usually associated with large companies in contract relations with farmers but in a scoping review of the literature, Liverpool-Tasie et al. (2020b) found this provision is common among SMEs as well. SMEs have the added advantage of being far more numerous than large contracting companies, and are often close to small farmers, clustered in small towns or secondary/tertiary cities near the farm areas.^5^

In output value chains, wholesale and logistics SMEs help small farmers directly with resource-provision arrangements. This was illustrated in the cases discussed above of traders in Asia and Nigeria, as well as cold storages in India.

In input and agricultural services value chains, wholesale and retail SMEs help small farmers as well. This is partly by solving the input access problem of farmers by bringing the fertilizer and seeds to farmers who do not have the time and means to go to towns to buy the inputs, such as in Nigeria (Liverpool-Tasie et al., 2019). This is also partly through provision of "outsourced mobile services". For example, in Indonesia, "sprayer-trader" teams provide land preparation, spraying, pruning, harvesting, and wholesaling mangos (Qanti et al., 2017). In Ethiopia, similar firms provide seed propagation, well and pond digging, spraying, land preparation, harvesting, and truck loading for vegetable farmers (Minten et al., 2020a). These services help farmers without the finances to invest in machines, the skills to use machines and other inputs, or simply the time to use in farming because of nonfarm employment.

## SMEs in the processing segment

The historical sequence of transformation of food processing in developing regions can be described by a J curve. Its horizontal axis is time (coincident with the sequence of stages of transformation from traditional to transitional to modern). Its vertical axis is the degree of concentration in the processing sector. The traditional stage featured micro enterprises in a broad informal sector and large parastatals in a limited formal, urban sector. The middle, transitional stage (generally after privatization of parastatals and market liberalization) featured an expansion of the processing sector with a proliferation of SMEs. This led to a de-concentration of the processing sector and thus a dip in concentration relative to the traditional (cum parastatals) stage. The modern stage (the right-most part of the J curve) featured competitive investment by foreign firms after FDI liberalization in the 1990s as well as competitive domestic investment which increased the share of food processing LEs, often at the expense of SMEs especially in categories like grains and edible oils where there are strong economies of scale in processing (Reardon, 2015).

Overlaying the 50-year trend of de-concentration then re-concentration phases of the J curve, the processing segment transformed with respect to its technology and its degree of processing. This involved first the rise of first-stage processing, or minimal processing such as grain flour and milk powder and edible oils, and then the rise of second-stage processing such as breads and noodles and packaged sauces, and ultra-processed foods such as cookies and other confection and sweetened beverages. We explore the stages in more detail below.

### The rise of “minimal processing” SMEs

In the traditional stage, little processed food was purchased. The great majority of food was processed and prepared at home. Emerging late in the traditional stage are hammer mills (as occurred in Africa in the 1970s/1980s) ushering in the spread of custom milling of grain and edible oil. There was strong demand by women to use these custom mills to avoid the onerous hand pounding of grain and tubers (averaging 4 h per day per woman in Africa in the early 1980s, see Barrett & Brown, 1994).

During the early transitional stage there is a steady rise of demand for purchased-processed food and the concomitant proliferation of processing SMEs producing minimally processed food such as bulk edible oil and flour. This was driven on the demand side by the increase of the opportunity cost of women’s time in cities and rural areas as women increasingly worked at least part time outside the home in rural nonfarm employment (RNFE) and on the supply side by the diffusion of processing equipment and technologies (Reardon et al., 2021).

The SME processors had by the mid and late transition stage largely shifted from custom milling, to sale of loose, unpackaged, and unbranded products, to sale of packaged and branded flour, rice, oil, milk, and so on, in small shops and emerging supermarkets. Examples of rapid proliferation of minimal processing SMEs shifting to packaged and branded products are found for wheat in Sri Lanka (Senauer et al., 1986), maize meal in urban Tanzania (Snyder, 2018) and rural Tanzania (Alphonce et al., 2019), teff and milk in Ethiopia (Minten et al., 2016a, 2020b), and rice in China (Reardon et al., 2010a).

By the modern stage, the SME minimally processed food share declines as large processors rise to dominate the market, relying on the advantages of economies of scale and widely recognized brands. For example, by the 2010s, approximately half of the rice in Beijing was bought from supermarkets and most of that was sourced from large mills; small mills had rapidly exited the sector over the 2000s in China (Reardon et al., 2010, 2014). While SME maize processors still dominated in Tanzania in the 2010s, large firms like Bakhresa dominated imported wheat milling (Snyder, 2018).

### The emergence of SMEs producing highly processed and prepared foods

In the early transition stage, SMEs produce unpackaged highly processed foods in the form of prepared snacks such as fritters and prepared dishes sold in kiosks at roadside or in markets. An example is the development over the decades in West Africa of SMEs making and retailing a prepared traditional dish with millet and fermented milk (such as degue in Ouagadougou, Burkina Faso in the 1980s (Reardon et al., 1989) and thiakry in Dakar in the 2010s (Chase-Walsh, 2018)). These are just examples of a wide variety of such prepared foods made and sold by SMEs in West African (Bricas et al., 1985) and many developing country cities to serve food-away-from-home (FAFH) demand created by men and women commuting to work outside the home, adolescents snacking near schools, and consumers at business districts and markets and transport hubs eating at street vendors at breakfast and lunch.

In the late transition stage, SMEs produce packaged high and ultra-processed foods such as chips, donuts, and cookies, and compete in these products with large enterprises, such as the regional multinational Bakhresa based in Tanzania (Reardon et al., 2021a). Initially these tend to be packaged and branded forms of traditional products, such as pickled sauces in India, and packaged “fura da yoghurt” in Nigeria (a millet/yoghurt dish packaged and sold in shops with refrigerators or at food service outlets, using yoghurt rather than the traditional version using traditional fermented milk). SMEs also clean and sort and package traditional grains and beans or cut up vegetables (ready to cook) sold in unbranded packages in wet markets.

Further along in the transition stage, SMEs begin to produce non-traditional products for urban markets, such as wheat bread in East Africa, the consumption of which surged driven by women's opportunity cost of time and commuting and the opening of many bakery SMEs (Kennedy & Reardon, 1994). As with minimally processed foods, highly processed foods made by SMEs shift from loose and non-branded (such as fritters on a tray) to being packaged and branded and sold in small shops. This is an important source of employment for women, requiring relatively little initial investment.

In the modern stage, the SME share falls as large enterprises emerge to dominate the high and ultra-processed food sector, again relying on economies of scale and widely recognized brands. For example, an illustration of the modern stage occurring in a specific product sector even though most of the food economy of a country is in the transition stage, is baked goods in Zimbabwe. Bakhresa (Tanzania) FDI bought the South African FDI firm Blue Ribbon Foods to lead the handful of bakery firms dominating that sector in the 2010s (Reardon et al., 2021a).

### The importance of “spontaneous clusters” of processing SMEs in the transition stage

Our studies (Table 1) tend to show that the proliferation of processing SMEs in the past several decades in SSA and South Asia often formed in “spontaneous clusters.” These clusters are sets of firms agglomerated spatially from common interest to be near sources of supply of raw materials (such as near a wholesale market) and near dense traffic of wholesalers buying for retailers and retailers who then disperse through cities.

For example, clusters of SME millers have formed around maize wholesale markets in Dar es Salaam in Tanzania. They benefit from ease of buying maize grain and of agglomeration to ease semi-wholesalers, retailers, and consumers access to their flour (Snyder, 2018). Clusters of teff millers, enjera (teff pancakes) processors, and SME retailers emerged rapidly in Addis Ababa in the 2010s. Traditionally consumers bought teff grain, cleaned it, custom milled it, and prepared enjera at home. But those practices declined in Addis Ababa in the 2010s, and consumers increasingly bought processed and prepared teff from SMEs. This development was facilitated by large investments (viewed in the aggregate over thousands of SMEs) by teff wholesalers and truckers, discussed above. In Nigeria, clusters of fish smokers and dryers SMEs formed near and in fish wholesale markets, fish farming areas, and river fishing landing areas in Kebbi State, Nigeria (Gona et al., 2018). A variant on the spontaneous clusters is indigenous “self-organized” cooperatives, often organized by product processor and trader associations. They most commonly appear in dairy, such as in Uganda (Van Campenhout et al., 2021) or Zambia (Neven et al., 2017).

Our working hypothesis based on the evidence we reviewed is that spontaneous clusters of SMEs are more important quantitatively, at least for SMEs and perhaps for the overall food economies, than are “state-managed clusters” (such as agroparks, agropoles, and special economic zones). Our hypothesis runs contrary to the great attention paid in policy debates to setting up managed clusters; we contend that at least equal emphasis should be placed on supporting the “endogenous and indigenous” emergence of spontaneous clusters.

## Impacts of midstream SMEs on employment, farm incomes, the environment, and consumption

There are a limited number of systematic surveys of wholesale and processing SMEs and few among those studies trace impacts on employment, farm incomes, the environment, and consumption. We use what evidence we found and illustrate pathways of impacts.

### Impacts of midstream SMEs on employment: levels and pathways

#### The importance of midstream SME employment in rural and urban areas

To explore impacts on employment we draw on Dolislager et al. (2020) which used LSMS survey data covering 178,794 households with 460,654 individuals in SSA (represented by Ethiopia, Malawi, Niger, Nigeria, Tanzania, and Uganda), Asia (Bangladesh, Cambodia, Indonesia, and Nepal), and Latin America (Mexico, Nicaragua, and Peru). Several of their results stand out.

First, what they refer to as "food systems employment" (in agrifood processing, wholesale, logistics, retail, and food service, mainly in SMEs as self- or wage-employment) is important in overall rural employment. It averages 20% of rural FTEs (24% in SSA, 18% in Asia, and 21% in Latin America). That is only modestly below the share of own-farming in total rural FTEs, averaging 29% (39% in SSA, 27% in Asia, and 16% in Latin America), and above the share of employment in farm wage labor, which averages only 9% of FTEs (3% in SSA, 13% in Asia, and 12% in Latin America).

Second, the food system employment impacts suggest inclusion of youth and women. (1) There is youth inclusion: in SSA, 24% of adults FTEs and 21% of youths' FTEs are in this employment; in Asia, 26% versus 32%; and in Latin America, 21% versus 23%. (2) There is female inclusion: over regions, the share of females’ total FTEs is 30%, versus only 19% for males. These findings coincide with SME enterprise studies for example in agro-processing in Ghana where Ampadu-Ameyaw and Omari (2015) find that women dominate the segment.

However, there is mixed inclusion of hinterland or lagging zones. Overall, rural peri-urban, intermediate, and hinterland zone households show similar shares for food system employment in total FTEs: 26%, 25%, and 22% respectively. However, in Asia and Latin America, food system employment was negatively correlated with distance from towns: in Asia, 20%, 12%, and 11% from peri-urban to hinterland; in Latin America, 26%, 13%, and 15% respectively. This suggests agglomeration effects near towns spurring wholesale and processing.

Third, they also found "food system employment" important in urban areas, averaging 25% of urban FTEs (31% in SSA, 27% in Asia, and 22% in Latin America). Note that the share in rural Asia was but 18% so this shows that a lot of the processing and wholesale is agglomerated in urban areas in Asia but less so in SSA. As in rural areas, in cities the reliance on food system employment is similar between urban youths and adults.

Most of this urban employment is in SMEs, at least in SSA and South Asia where the penetration of large-scale processing firms as well as large wholesale and logistics firms is still slight. For example, in Ghana, in a survey of 282 agro-processing firms, Afful-Koomson et al. (2014) (cited by Owoo & Lambon-Quayefio, 2018) found that 97% of the agro-processing firms were “very small or small SMEs,” and only 3% are medium sized firms (although the latter counted 30% of total processing employment). In Ethiopia, medium and large-scale agro-processing firms are defined as those that employ 10 or more people and use electricity-driven machinery. They represent only 1% of all agro-processing firms and 7% of their employment, with the rest accounted for by small firms (Minten et al., 2016a).

#### Conditioners of employment and income effects of SMEs

First, technological change in SMEs can increase the employment they induce. An example is the shift in enjera processing from traditional wooden plates to new electric plates (mitads) which led to a higher capacity of the SMEs and better quality of the product, and a marked increase in employment in the sector (Minten et al., 2016b).

Second, there is emerging evidence that SMEs “climb the value ladder” to achieve higher incomes through branding and product differentiation. For example, Reardon et al. (2014) show that in Bangladesh, China, India, and Vietnam, rice mills have gone from custom milling (where customers bring paddy to be milled) to packaging and branding their rice.

Third, SMEs’ growth creates multipliers in other midstream SME segments thus further spurring employment. An example is the extensive use that urban maize traders make of 3PLS SMEs in Nigeria, moving some 80% of maize with these services (Liverpool-Tasie et al., 2017).

Fourth, SMEs produce time-saving goods and services that liberate especially women but also men to pursue more employment (or leisure time). Above we noted that African females used to spend 4 h a day pounding staples, time that was saved by their being able to buy flour from SMEs. Liverpool-Tasie et al. (2016) show that in Nigerian villages greater access to processed food is associated with more female employment.

#### SMEs are generally more labor-intensive per ton of output than large enterprises

First, a common finding in the limited number studies comparing midstream SMEs versus large enterprises is that that SMEs are less capital-intensive. Examples of this include the positive correlation of the capital/labor ratio shown for small versus large enjera enterprises in Minten et al. (2016a), and a positive correlation between capital/labor ratios and rice mill scale in India, China, and Bangladesh (Reardon et al., 2014).

Second, the corollary is that it appears that usually SMEs are more labor intensive (in terms of labor/output ratios). For example, for China, Reardon et al. (2012) show that SME rice mills used 1.5 times more labor per ton of rice sold than large mills. For Tanzania, Snyder et al. (2017) shows that as total milling enterprise receipts increase 300-fold from very small to very large mills, the productivity of FTEs of labor to produce nearly 500 dollars of maize flour drops 140-fold.

However, larger firms often have a higher capacity (capital) utilization rate and its correlate, the profit rate. This was shown for example in the rice mill survey results presented in Reardon et al. (2014) for China, India, and Bangladesh. The country studies showed that larger mills have a wide procurement area that allows nearly continuous purchase of paddy from different production regions, while small village mills relied on the local paddy production and thus had higher fixed costs per unit of output. This is likely a reason for the rapid disappearance of village mills and the "flattening" of the employment impact of processing growth over time, such as in India. This appears to also be the case for wholesaling, as shown in studies of shrimp traders in Indonesia (Yi & Reardon, 2015) and produce traders in the former Zaire (Minten & Kyle, 1999).

Third, another factor that reduces the share of smaller SMEs (and thus by corollary reduces the SME labor impact over time) is the exit of smaller SMEs that cannot compete in product differentiation. For example, Reardon et al. (2014) show in Bangladesh that the larger the mill, the more likely the mill is to have color sorting and rice polishing equipment lines which increase the rice grade; this is a significant threshold investment not feasible for small mills.

### Pathways of impacts of midstream SMEs farmers’ incomes

First, SMEs in the midstream of output value chains are the actors closest upstream and, with input retailers, downstream from farmers. SMEs have been crucial in creating a “vent-for-surplus” and value addition which facilitates farmers’ commercialization. SMEs signaling quality requirements and opening markets and services for high-value products help small farmers to shift from low-value basic food grains; an example is the rise of cold storages in the poor state of Bihar, India (Minten et al., 2014b). Large processors and supermarket collection centers play only a small role as market outlets for small farmers in the transition stage, and tend to limit their sourcing to only the most favorable areas (Barrett et al., 2012).

Second, discussed in Sects. 3 and 4 in detail but noted here for completeness, we signal two service provision avenues of SMEs' helping farmers. (1) SMEs in the midstream of output value chains help small farmers indirectly by establishing resource-provision relations with farms that bring farmers services such as logistics and inputs and credit. (2) Midstream SMEs provide agricultural services such as combine harvesting and marketing as well as training.

Third, SMEs create opportunities for rural non-farm income generation (both in self-employment and in wage-employment) by rural households (with farmland and also the landless). This income is a major source of cash for farm investments and technology adoption often much more important than credit (Reardon et al., 1995; Adjognon et al., 2017). As we noted above, midstream employment in the AVC is an important part of rural nonfarm employment.

### Pathways of impacts of midstream SMEs on the environment

First, midstream SMEs’ technological choices can directly affect the environment. For example, over ten years Ethiopian grain trucking SMEs nearly doubled the average size of their trucks. This led to a 50% decline in transport costs with lower fuel use per ton of grain moved, despite the elimination of fuel subsidies (Minten et al., 2016a).

Second, midstream SMEs’ can affect the environment via their effects on farms. Farmers' commercialization via wholesale SMEs often increases farms profits and induces intensification via increased use of productivity enhancing inputs such as fertilizer, soil integrity amendments such as manure, and soil conservation via bunds and terraces, such as in Rwanda (Clay et al., 1998). That intensification can in turn reduce pressure to extensify into the commons (Angelsen & Kaimowitz, 2001).

However, commercialization-induced intensification can also induce excessive use of pesticides and fertilizer with attendant pollution of rivers and groundwater, such as occurred in Southeast Asia (Pingali & Rosegrant, 1995), or siltation from aquaculture and effluents of manure from pig and chicken production. Sometimes SME chemical wholesalers directly induce as much use as possible which can be excessive; for example, Al Masud et al. (2020) showed that in Bangladesh poultry dealers provided credit to small poultry farmers for day-old chicks and feed but bundled antibiotics sales to the farmers in the arrangement, inducing over-use.

Yet sometimes SMEs can mitigate farmers' over-use of inputs even while selling them inputs. For example, Liverpool-Tasie et al. (2020a, b, c) noted that agro-dealer SMEs sometimes bundled training in proper input use with the provision of chemicals.

### Impacts of midstream SMEs on consumers

#### Midstream SMEs affect consumer food security

First, the extent to which SMEs pass on farm productivity gains to consumers, and input cost reductions to farmers, is a condition of transaction costs SMEs face (such as road quality and bribes), the efficiency of the SMEs, and whether they engage in non-competitive behavior. The impact can be positive, such as we illustrated above for Ethiopia where wholesale and transport SMEs reduced wholesale and retail margins and transport costs cheapening teff products for consumers.

Second, SMEs' investments can reduce waste in value chains, increasing food access for consumers. For example, cold storage SMEs in Bihar, India, displaced traditional on-farm storage and thus reduced wastage rates (Minten et al., 2014b). Processing SMEs reduce waste, such as those that dry tomatoes and dry and smoke fish.

Third, SMEs can de-seasonalize consumer food access while raising prices farmers receive, as we discussed above for the case of potato cold storage SMEs in India. Fish drying and smoking SMEs are important in Nigerian fish consumption volumes as well as reduced seasonality relative to the fresh fish market (Liverpool-Tasie et al., 2021) and for employment in the poorer north of Nigeria (Gona et al., 2018).

#### Midstream SMEs affect food quality/nutrition for consumers

First, SMEs are important as suppliers to satisfy the consumer demand for nutritious foods. For example, about 75% of mangoes produced in Indonesia are consumed in urban areas, with nearly all of this supply chain operated by SME traders and retailers (Qanti et al., 2017); dairy SMEs in processing, logistics, and wholesale are the mainstay of milk supply to Uganda (van Campenhout et al., 2021); the great majority of fish and chicken supply to urban areas of Nigeria (where the great majority of these products are consumed) are mainly operated by SMEs (Liverpool-Tasie et al., 2017); SMEs are the mainstay of the supply chain functions of transport, packing, and wholesale of vegetables to Addis Ababa (Minten et al., 2020a, b).

Second, SMEs produce and sell nutritious traditional meals especially in urban areas (Reardon et al., 2021a), such as millet and dairy dishes in Senegal (Chase-Walsh, 2018) and in Burkina Faso (Reardon et al., 1989) and teff enjera in Ethiopia (Minten et al., 2016a). They make and sell milled nutritious flour such as pulses and coarse grains as weening foods in Tanzania (Snyder et al., 2015).

However, a subset of SMEs that produce and market processed foods manufacture ultra-processed foods that are bad for consumers’ health, such as a range of snack food SMEs in Africa (Reardon et al., 2021a). Moreover, most consumers’ purchases of ultra-processed foods are made in small traditional shops, mainly because they still dominate food retail such as in Kenya (Demmler et al., 2018).

#### Midstream SMEs affect food safety for consumers

First, SMEs' food handling conditions the safety of foods for consumers. For example, Sanou et al. (2021) demonstrate how maize traders respond to consumer preferences via higher willingness to pay for maize safety attributes their clients exhibit preferences for.

Second, as noted above, there has been a rapid recent emergence of packaging and branding by SMEs in the midstream in developing regions. This is sometimes used to signal safety (such as cleanliness) and permit quality differentiation. However, the labeling does not always guarantee safety or even improved quality as there are usually poor regulatory systems in developing countries such as lax or missing labeling and food safety regulations. For example, Minten et al. (2013a, b) note that branding of makhana (an aquatic crop made into a snack or a high-protein low-fat food) spread recently in India; but that did not guarantee that the quality or safety of the product was superior to non-branded makhana due to lack of regulation.

Third, as expected, there is uneven adoption of food safety measures by SMEs in the midstream. Strict public standards as well as even more demanding private standards of large food manufacturers and supermarket chains can result in exclusion of food processing SMEs that cannot meet the standards, such as in Brazil for the cases of milk and shredded coconut (Reardon & Farina, 2002). The needed “threshold investments” in skilled labor practices, equipment, and building refitting are sometimes beyond the means of SMEs.

## Conclusions

In this paper we examined the development of SMEs in the midstream segments (wholesale, logistics, and processing) of the food output and farm inputs value chains in developing regions. We situated that development in the context of the stages of transformation of AVCs from traditional to transitional to modern. We focused on the growth and importance of SMEs in the long transitional stage, in which most of SSA and South Asia now are, as well as parts of Southeast Asia and Latin America. We reviewed evidence for and noted impact pathways of those SMEs on employment, farmers, consumers, and the environment. We relied on a broad review of the findings from our own surveys of thousands of SMEs mainly in SSA and Asia, and the findings of studies by others. Due to space constraints, we did not attempt to review the literature on the constraints SMEs face or policies and programs aimed at regulating and assisting SMEs. Following are the main findings and implications.

First, there has been a rapid expansion of SMEs in the midstream segments of AVCs in the developing regions, paralleling the rapid growth of the volume and length of AVCs. The latter has been spurred mainly by the "demand pull" of urbanization accompanied by the facilitating influence of development of public infrastructure, in particular wholesale markets and roads.

Second, the midstream SMEs taken as a whole handle roughly two-third of the food consumed in the developing regions; the rest is handled by modern firms and a minor share is subsistence consumption. Thus, midstream SMEs are crucial to the food security of billions of persons in these regions. Their share in the food economy is roughly similar to farms themselves, yet midstream SMEs are often "hidden" from the food security policy debates. They are often considered a "missing middle", but we argue that they are already a dynamic and massive presence. They should be termed the "hidden middle," as they are not missing but are merely usually hidden from the debate.

Third, we found that midstream SMEs condition employment, farm incomes, the environment, and the food prices, food safety, and food quality facing consumers. It appears that often the effect is important and positive. The employment by SMEs forms roughly a fifth to a quarter of rural and urban employment in these regions and is disproportionately important as jobs for women and youths.

Fourth, and crucial to the policy implications here, is that we found that where the enabling conditions (such as wholesale markets and good roads) are present, SMEs emerge and grow rapidly, often in "spontaneous clusters" rather than in the "managed clusters" that dominate the policy debate. Emblematic are the cases of: (1) the wholesale, logistics, and processing SMEs involved in the teff value chains feeding Addis Ababa, Ethiopia, its leading staple food; over just a decade these firms tripled in number (and more than tripled in total volume distributed), they made investments in their own capacity that drove down transport costs and supply chain margins; (2) the potato cold storage SMEs in Agra, India, feeding Delhi its leading staple vegetable; over a decade these firms also tripled, making investments that reduced potato supply seasonality to cities and increased farmer incomes; (3) the wholesalers and input firms in the aquaculture supply chain in Bangladesh; over just a decade they tripled in numbers and more than tripled in volumes, facilitating a boom in fish supply to cities and creating substantial employment. These three cases represent many others we identified, and are important not only for the dynamism they portray, but because in none of the cases did any donor, NGO project, subsidy or direct government action play a significant role. In all three cases however the governments' provision of wholesale markets and roads and electrification were crucial. When the conditions were present, the SMEs spread and grew to meet the rapidly rising urban demand, creating rural and urban livelihoods.

The policy implications of the above four findings are that multilateral agencies, donors, governments, and NGOs do not need to “kickstart” SMEs in the midstream and downstream of food supply chains. There is no need to put in place services that these firms are already providing and thus “reinvent the wheel.” Rather, support should focus on leveraging the rapid growth of SMEs already in place or emerging, and addressing their constraints to faster growth and better impacts. Donors and governments should not waste resources trying to “organize” SMEs into cooperatives or managed clusters, force them into formal patterns, or try to “create them” with programs that set up firms. Extrapolating from our findings that infrastructure coupled with urban demand was usually the key enabler, we recommend a focus on infrastructure provision.

While we did not analyze the constraints to SMEs, our prior work has pointed to SMEs' being constrained by poor wholesale markets, bad roads, spotty electrification, and bribes on the roads. We believe that addressing these should be the first and main priority.

Moreover, there is a need to address bad policies and regulations that constrain SMEs. Bribes are a kind of informal regulation or informal tax that the government at various levels imposes on SMEs and are a key regulation problem. Other bad regulation includes needless red tape for redundant certifications, confusion and ambiguity in zoning and premises, and in times of crisis like COVID-19, deleterious limitation on mobility (such as shown in Nigeria, Liverpool-Tasie et al., 2020a).

Finally, there are missing or poorly implemented food safety and hygiene regulations that make SMEs with unsafe practices a danger to consumers and a constraint to themselves. This also extends to the need for truth in labeling regulations. There is a role for education of SMEs in food safety standards to complement regulation.

## Footnotes

1 We use "SME" because it is commonly used in international debates. They are in two categories: (1) micro-enterprises, often home-based, operated by one or two family members, in rural and urban areas; (2) small and medium enterprises usually non-home based, operated by family but also non-family hired workers, and often in the intermediate or peri-urban rural areas and cities. Sometimes the term “MSME” is used for this set that includes micro firms, but we eschew MSME because it is not commonly used in the literature.

2 Urban populations increased fivefold from 1970 to 2020 in developing regions (53 to 469 million in SSA; 210 to 1210 million in developing East Asia; 133 to 648 million in South Asia; 164 to 529 million in LAC; https://data.worldbank.org/indicator).

3 Examples are the many thousands of rural “haats” in India, managed by villages (Government of India, 2001).

4 https://eros.usgs.gov/westafrica/case-study/urban-sprawl-ibadan-savanna-and-forest-habitat.

5 It is possible that small farmers that shift from relations with SMEs to contract relations with large companies do not experience an advantage and may even lose some of the benefits of dealing with SMEs; this has not yet been explored empirically to our knowledge.
